# Designing broad-spectrum anti-HIV-1 gRNAs to target patient-derived variants

**DOI:** 10.1038/s41598-017-12612-z

**Published:** 2017-10-31

**Authors:** Will Dampier, Neil T. Sullivan, Cheng-Han Chung, Joshua Chang Mell, Michael R. Nonnemacher, Brian Wigdahl

**Affiliations:** 1 Department of Microbiology and Immunology, Drexel University College of Medicine, Philadelphia, PA USA; 2 Center for Molecular Virology and Translational Neuroscience, Institute for Molecular Medicine and Infectious Disease, Drexel University College of Medicine, Philadelphia, PA USA; 3 School of Biomedical Engineering and Health Systems, Drexel University, Philadelphia, PA USA; 4 Center for Genomic Sciences, Institute for Molecular Medicine and Infectious Disease, Drexel University College of Medicine, Philadelphia, Pennsylvania USA; 5 Center for Advanced Microbial Processing, Institute for Molecular Medicine and Infectious Disease, Drexel University College of Medicine, Philadelphia, Pennsylvania USA; 6 Sidney Kimmel Cancer Center, Thomas Jefferson University, Philadelphia, PA USA

## Abstract

Clustered regularly interspaced short palindromic repeats (CRISPR) CRISPR-associated protein 9 (Cas9), including specific guide RNAs (gRNAs), can excise integrated human immunodeficiency virus type 1 (HIV-1) provirus from host chromosomes. To date, anti-HIV-1 gRNAs have been designed to account for off-target activity, however, they seldom account for genetic variation in the HIV-1 genome within and between patients, which will be crucial for therapeutic application of this technology. This analysis tests the ability of published anti-HIV-1 gRNAs to cleave publicly available patient-derived HIV-1 sequences to inform gRNA design and provides basic computational tools to researchers in the field.

## Introduction

The clustered regularly interspaced short palindromic repeats (CRISPR) CRISPR-associated protein 9 (Cas9) system is a gene-editing system that uses a guide RNA (gRNA) to bind a specific DNA target and subsequently induce double-stranded breaks (DSBs), catalyzed by the gRNA-bound endonuclease Cas9. The CRISPR/Cas9 system has been widely implemented as a novel strategy for combating viral infections and inactivating specific pathogens and cellular genes. The long terminal repeat (LTR), has been the primary target for excising almost the entire HIV-1 provirus because there are two identical copies located at each end of the integrated proviral genome, so DSBs at both sites facilitate the removal and subsequent degradation of the intervening HIV-1 open reading frames (ORFs)^1–14^. To date, anti-HIV-1 gRNAs have been shown to be efficient at either activating the LTR from latently infected cells as a novel “Shock and Kill” strategy, disrupting the function of particular HIV-1 genes, or excising the HIV-1 provirus from cells with no detectable off-target mutations or cell toxicity (Table 1). However, HIV-1 displays a high level of genetic diversity across the genome both among and within patients. Before CRISPR/Cas9 can become a viable therapy for HIV infection/disease, it is necessary to evaluate how well anti-HIV-1 gRNAs can cleave polymorphic viral genomes from patient samples.

**Table 1:** Regional distribution of anti-HIV-1 gRNAs and the number of those with broad-spectrum activity.

Region	Total	EF50	EF90
Env	14	5	2
Gag/Pol	57	32	0
LTR	148	89	2
Nef	1	1	0
Rev/Env	3	3	0
Tat/Rev	3	2	0
Vif	2	0	0
Vpr	1	0	0

This table displays the number and region of anti-HIV-1 gRNAs. EF50 represents the number of gRNAs that are predicted to cleave at least 50% of patients and EF90 represents the number of gRNAs that are predicted to cleave at least 90% of patients.

Targeting regions of high sequence conservation is one strategy for addressing the highly mutable HIV genome. Conserved regions within a highly variable genome are ideal targets for gene-editing strategies, since mutations that escape the therapy will likely lead to low viral fitness. Recent experiments by Wang *et al*. has demonstrated that viral escape takes much longer when gRNAs target conserved versus non-conserved regions^15^. In addition, the time until viral escape can be significantly increased when multiplexing two gRNAs^12^.

Extensive research has gone into determining the parameters responsible for gRNA targeting and subsequent binding and cleavage^16–18^. The MIT CRISPR Design Tool is commonly used to design gRNAs to target particular DNA sequences, along with a measure of possible off-target cleavage (http://crispr.mit.edu). Using experimental data, the group developed a penalty function that describes the CRISPR/Cas9 activity with a position-specific mismatch penalty that captures the inherent promiscuity of Cas9 targeting^17^. The score derived from the MIT scoring function, termed here as the Activity Score (AS), can be thought of as the probability (from 0-1) that a particular gRNA will cleave a target sequence. The AS inherently does allow for mismatches between the query gRNA and the target sequence but penalizes mismatches proximal to the protospacer adjacent motif (PAM) more heavily than those distal to the PAM. The MIT tool uses an aggregate of the AS calculated across the human genome to evaluate the risk of potential off-target cleavage (termed here as MIT-off). In the context of anti-HIV-1 gRNAs, this type of analysis has thus far only been used to capture the “host” side (*i.e*. the human genome reference) of the question based on a single input sequence.

We have adapted the AS to predict the likelihood of double-stranded cleavage of patient-derived HIV-1 sequences. Using this tool, we present an *in silico* evaluation of how effective each of the 229 anti-HIV-1 gRNAs designed to date perform when evaluated using current knowledge of the genetic diversity of HIV-1. It should be noted that we gathered all published anti-HIV-1 gRNAs regardless of their experimental use, and we understand that not all gRNAs were designed to target patient viral quasispecies. Nonetheless, they were included in this analysis to determine their broad-spectrum capabilities. In order to evaluate each gRNA’s performance, a pipeline incorporating the AS was developed to calculate an HIV-1-specific on-target score and to determine the fraction of known patient-derived HIV-1 sequences that would be cleaved by a particular gRNA. This pipeline can be used to filter out gRNAs that may not be effective for a majority of patients.

## Results

### *In silico* evaluation of current anti-HIV-1 gRNAs for potential HIV-1 cure strategies

To evaluate the *in silico* effectiveness of the 229 published anti-HIV-1 gRNAs, the gRNAs were tested against all 390,290 subtype B patient-derived HIV-1 sequences from the Los Alamos National Laboratory (LANL) HIV sequence database (https://www.hiv.lanl.gov/content/sequence/HIV/mainpage.html) spanning the entire viral genome. We designed a gRNA pipeline that calculates an AS against all collected sequences which overlapped their respective target regions^1,3,4,6–15,19–31^. These scores were binarized using an initial AS cutoff value of 0.75 and then were screened to determine the predicted percentage cleaved (PPC) by specific gRNAs. It is important to note that the AS used to calculate PPC is distinct from that returned by the MIT CRISPR Design Tool denoted here as MIT-off. Instead, the AS produced represents the likelihood of a single gRNA cleaving a target HIV-1 sequence (“HIV on-target”), while the MIT-off indicates the likelihood of particular gRNAs to induce an off-target DSB event in the human genome. To facilitate this comparison, both the HIV-1 PPC and the MIT-off for relevant gRNAs are indicated (Supplementary File 1).

**Figure 1:**
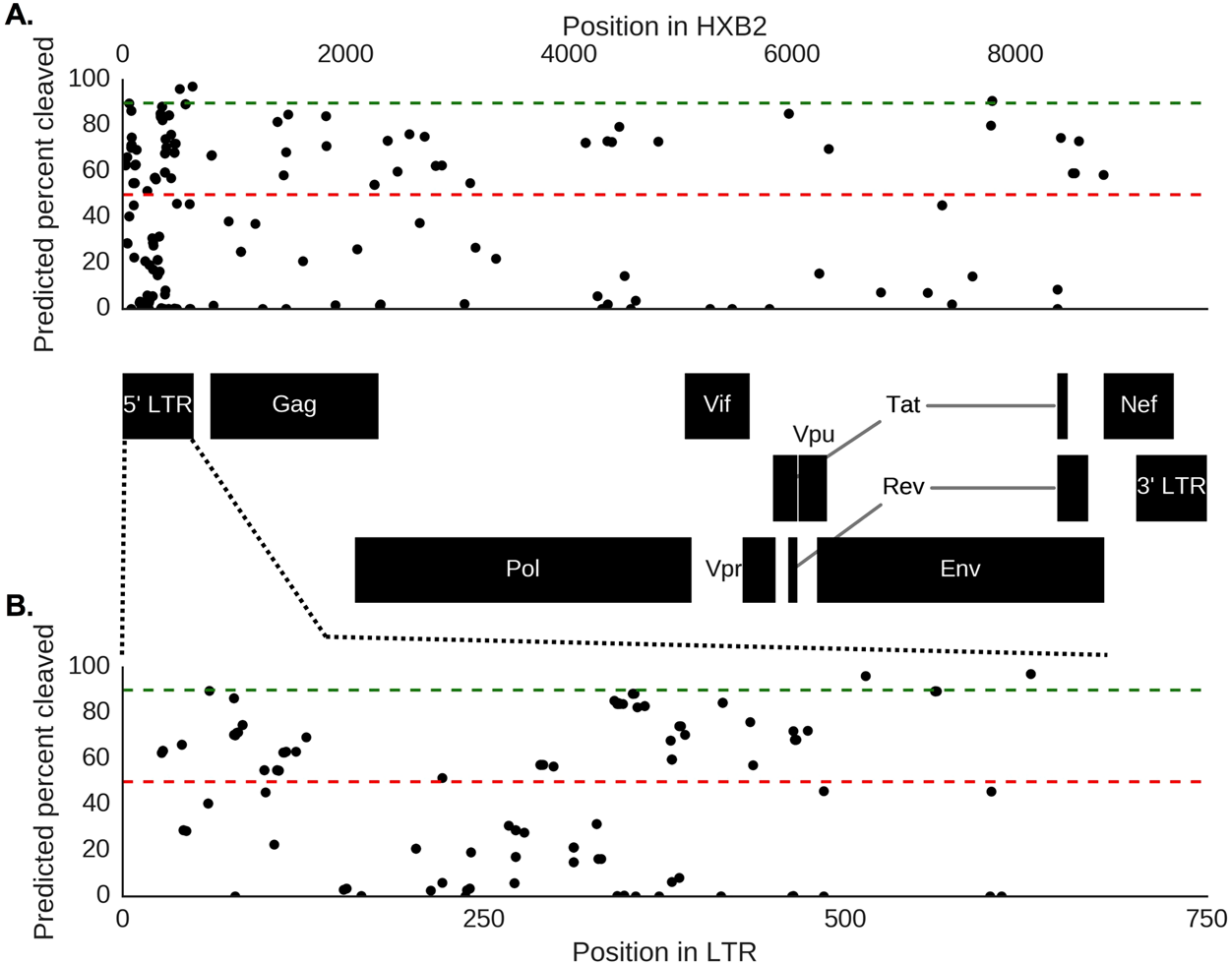
*In silico* evaluation of current anti-HIV-1 gRNAs for HIV-1 CRISPR therapeutic strategies are unable to the target genetic variants in patient-derived HIV-1 sequences. All published anti-HIV-1 gRNAs were individually screened against HIV-1 sequences in LANL to determine their efficacy. (**A**) The scatter plot depicts the percentage of patient samples predicted to be cleaved by published gRNAs (PPC) throughout the entire HIV-1 genome. Each dot represents a particular anti-HIV-1 gRNA in relation to PPC. The HXB2 genome position coordinates and map are displayed on the top and bottom of the x-axis, respectively. (**B**) Since LTR specific gRNAs are ideal for excision therapy, we represented them again in relation to the LTR alone. LTR-specific gRNAs are shown where each dot represents the mid-point of the gRNA targeting position on HIV-1 genome. Red lines and green lines in (**A**) and (**B**) represent 50% and 90% predicted percent cleavage, respectively.

Published anti-HIV-1 gRNAs had highly variable predicted effectiveness at cleaving patient-derived HIV-1 sequences (Fig. 1A). Of all the anti-HIV-1 gRNAs examined, only 57.6% (132/229) were predicted to cleave more than 50% of the patient-derived sequences, and only four gRNAs were predicted to cleave more the 90% (Table 1) and bolded in (Supplementary File 1). LTR-targeting gRNAs also had highly variable PPC values, with gRNAs between positions 150–350 showing lower predicted targeting efficiency than other regions in the LTR (PPC less than 50%), reflected by the higher genetic diversity of this region (Fig. 1B). It is important to note that some of the gRNAs with poor HIV-1 PPC had 100% complementarity with the laboratory or reference strains they were designed against and have shown activity in their respective assays, but unfortunately these are not representative of sequences from patients and as such have low PPC values (Supplementary File 1) as would be predicted.

We next performed a sensitivity analysis to determine the effect of different AS cutoffs on the estimates of the HIV-1 PPC of each gRNA. This allowed us to determine the predicted effectiveness of CRISPR/Cas9 variants which may have different homology requirements. We attempted to capture this by calculating what the AS cutoff would have to be to match 50% of patients (AS50) (Supplementary File 1). In other words, we reduced the homology requirement of the gRNA to target matches such that at least 50% of the patient-derived sequences from LANL would be effected. The results indicate that 17% (39/229) of the tested gRNAs would require an AS <0.1 and 35% (82/229) would require an AS <0.5 to cleave 50% of patients. Conversely 58% (133/229) of the gRNAs tested would pass the 50% patient level if the AS cutoff was greater than the expected 0.75 cutoff. For gRNAs that still didn’t cut patient sequences when the AS was reduced, it was more often due to a loss of the PAM site in patient sequences rather than due to a loss of the gRNA target itself.

### Determining entropy of LTR gRNA target sites of patient-derived subtype B sequences

**Figure 2:**
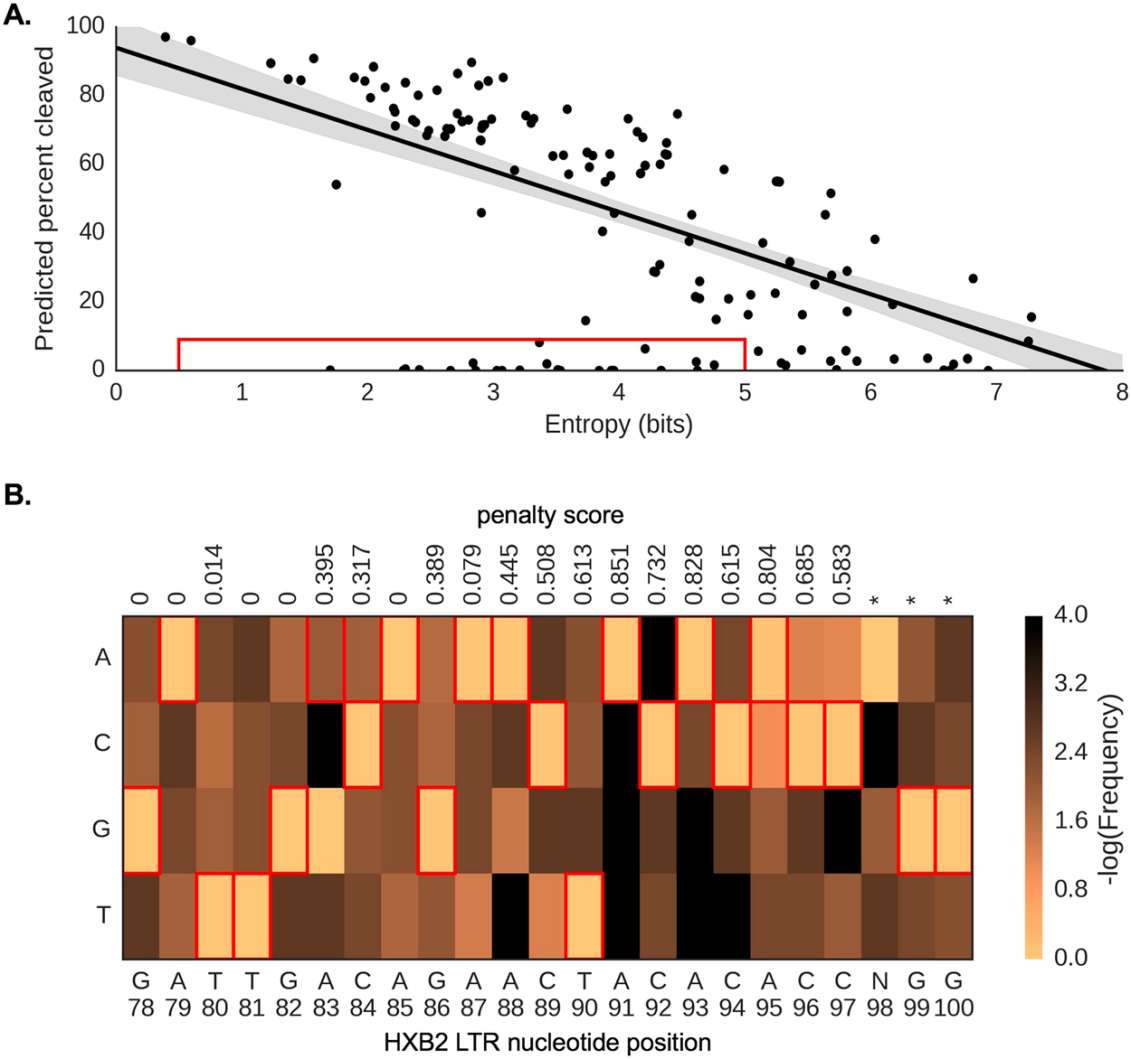
Some gRNAs targeting low entropy regions are still unable to cut patient-derived sequences due to genetic variation. All published anti-HIV-1 gRNAs were individually screened against HIV-1 sequences in LANL to determine the entropy of the gRNA target site and their predicted percentage of patients cleaved (PPC). (**A**) With increased entropy, there is a decrease in PPC. Each point indicates the 20-mer entropy and PPC. The linear trend-line has been shown in black with the 95% confidence intervals indicated as gray shadows. The red box indicates gRNAs that target low entropy regions but still have a low percent cleaved. (**B**) Low entropy has been shown to be a necessary, but not sufficient, factor in anti-HIV-1 gRNA design. The heat map represents the information content across the LTR-2 gRNA with the red boxes indicating intended target sequence. The position-specific mismatch penalty scores have been indicated across the top of the figure and the intended targeting position in the HXB2 genome has been denoted across the bottom.

Some of the more recent anti-HIV-1 gRNAs have been designed to account for HIV-1 genetic variation by using the LANL database to target regions of the HIV-1 genome with low Shannon entropy^12,15^. While this measure is ideal for determining the conservation of a region and the potential for viral escape from the therapy, Shannon entropy cannot directly account for the efficiency of gRNA cleavage the way that the AS does^12,15^. This is important because the AS allows mismatches of the gRNA to the target sequence at particular positions, generally it is more tolerant to those most distal from the PAM. Thus for all of the 229 gRNAs, the relationship between HIV-1 PPC and the Shannon entropy of the target regions was examined (Fig. 2A). Overall, lower entropies corresponded with higher PPC, showing a general linear relationship; however, due to the position-specific mismatch penalty inherent in the AS, there were significant outliers (denoted in red box, Fig. 2A).

Surprisingly, some gRNAs designed to target conserved regions with low diversity were still predicted to cleave only a minority of patient-derived sequences (Fig. 2A red box). This is because the target HIV-1 sequence used for designing these gRNAs contains uncommon variants in the LANL patient-derived sequences. This is likely due to experimental design choice to use a common laboratory or reference strain for developing the technology and not specifically for the purposes of patient treatment. The gRNA LTR-2 that targets HXB2 between coordinates 78 and 97 is an ideal example (Fig. 2B)^23^. This region has a low Shannon entropy in patient-derived sequences but the engineered gRNA targets an LTR sequence which contains a G83A mutation relative to patient sequences^23^. In this way, the researchers developed a gRNA to ask important experimental questions however, it could not be directly translated into a therapeutic vector. If the G83A mutation would have been identified and addressed, it would bring the effectiveness of this specific gRNA up to a 93% predicted cleavage rate, while possibly maintaining the low escape rate observed when targeting a similarly conserved region^23^. Examining all 229 anti-HIV-1 gRNAs, 89% (195/229) were within 2 edit distances from the patient consensus sequence and as such could be easily adjusted to match patient samples. This has indicated that the current anti-HIV-1 gRNAs are specific for their intended assays and have the potential to be redesigned to address patient-derived sequences if desired in a number of therapeutic avenues.

## Discussion

There has been significant progress within the field of HIV-1 cure strategies using CRISPR/Cas9. The field has demonstrated the efficacy of CRISPR/Cas9 in inducing insertions and deletions (InDels) into the HIV-1 genome, removing the entire genome, and reactivating the virus from latency^1–15,19–31^. However, only one study thus far has functionally demonstrated this in primary cells from HIV-1-infected patients and in that specific instance looking at a small patient population; 4 patients^8^. Therefore, this analysis was designed and performed to understand the *in silico* efficacy of current anti-HIV-1 gRNAs on patient-derived sequences to better inform gRNA design for *in vitro*, *in vivo, and ex vivo* experimentation. The above results show that it is crucial to consider not only the genetic diversity of the HIV-1 genome but the large number of specific variants that exist within the patient population when designing anti-HIV-1 gRNAs for therapeutic avenues.

Recent studies have shown the patient-derived HIV-1 sequences displayed a measureable and substantial amount of genetic variation even on stable, uninterrupted HAART with region-specific as well as patient-specific mutation rates, indicating that certain regions may be more easily targeted than others^2,32–35^. As is demonstrated in the cross-sectional data analysis performed herein, the same difficulty with target variation will be encountered within a patient. The problem of targeting the viral quasispecies is not limited to subtype B HIV and will likely be a factor for other subtypes as well. This is further exemplified by studies from Bialek *et al*. that have shown their anti-HIV-1 gRNAs function differently across different subtypes when considering a range of mismatches from LANL derived sequences^19^.

While only a handful of existing gRNA designed are predicted to overcome the genetic variation in patient-derived sequences, many of the gRNAs included in this analysis were designed with consideration towards the conservation of the gRNA to the targets and avoided targeting host transcription factor binding sites for many excision/InDel studies. This is further exemplified when targeting conserved versus non-conserved regions and the emergence of viral escape^15^. This escape can be progressively circumvented by multiplexing multiple gRNAs into a combination therapeutic agent^12^. In addition, there is some evidence that excision of the entire genome may not occur as frequently as a single disruption and incorporation of InDels in the LTR^8,12^. Therefore, a valid strategy could multiplex many gRNAs targeting multiple conserved regions across the proviral genome. However, broadly acting gRNAs should not be designed at the expense of an increase in off-target events. Current off-target functional techniques such as GUIDE-Seq, CIRCLE-Seq, and others have not been published in relation to the HIV-1 field but could greatly enhance our understanding of gRNAs with respect to therapeutic potential outside of broad acting activity against many patients^36,37^.

This report demonstrates how far the field of CRISPR/Cas9, with respect to HIV eradication, has come in a short amount of time and is intended to motivate the field moving forward. While understanding the diversity between infected individuals may be technically and financially cumbersome, it may be required to globally cure HIV-1 utilizing this type of technology, which exists as many genetically different subtypes. To this end, we have released a basic set of tools that can be used to examine the effectiveness of gRNAs against a genetically diverse target like HIV. It will also be important to balance the need of targeting laboratory-strains for rapid and reproducible experimental results against sequences from HIV-1-infected patients. With a greater wealth of patient-derived sequencing data and understanding the biology of the CRISPR/Cas9 system, the hope of having a HIV-1 cure therapy is one step closer to a reality.

## Methods

### LANL sequence query

To determine the PPC potential of patient sequences from the LANL database by published anti-HIV-1 gRNAs (gRNAs published as of 07/31/2017), selected regions of the HXB2 genome were downloaded as of 1/13/2017 (https://www.hiv.lanl.gov/content/sequence/HIV/mainpage.html). Using the LANL database tools, the selection was limited to North American subtype B samples originating from a patient. Only sequences of at least 100 bp and less than 1% ambiguous nucleotides were preserved for subsequent analysis. Alignments were performed using the LANL database tools. Due to the limitations of the LANL toolset the sequences were collected and aligned across 14 roughly 500 bp regions: 1–700, 700–1150, 1100–1950, 2000–2500, 2500–3000, 3200–3500, 4100–4900, 5200–5600, 5700–6100, 6200–6900, 7100–7500, 7500–7900, 8300–8900 and 8500–8800 spanning the HXB2 genomes (K03455). In total, this study used 390,290 sequences from the LANL database. These sequences have an average length of 390+/−166 bp.

### Activity Score calculation and on-target pipeline

The scoring of each gRNA against a potential sequence was performed using the MIT scoring function from Hsu *et al*. shown in Equation 1 ^17^. In brief, this methodology assigned a position-specific mismatch penalty to mismatches across the protospacer with those proximal to the PAM sequence being more deleterious than those distal to the PAM. A weighted average along with a dampening score was used to calculate a final score. Each gRNA was scored using the following algorithm to: (1) find all sequences from the LANL which overlap the target region, requiring 10 bp flanks; (2) apply the scoring function across all gRNA-target pairs in the ungapped sequence; (3) report the highest score for each sequence and indicate whether there was an adjacent NGG sequence; and (4) use a cutoff of AS >0.75 to define whether a gRNA would cleave a given sequence. If the adjacent NGG PAM sequence was missing the AS was set to 0 for that particular sequence.

1 $$AS=\prod _{e\in M}(1-W[e])\ast \frac{1}{\frac{19-\bar{d}}{19}\ast 4+1}\ast \frac{1}{{n}^{2}}$$

The Activity Score as determined by Hsu *et al*.^17^. *e* represents the position in the gRNA starting at the PAM distal end. *W[e]* is a penalty function that evaluates to 0 if position *e* is a match between the gRNA and the target and a position-specific penalty M in the event of a mismatch at position *e*. Position-specific penalty scores M are shown in Fig. 1D. $\bar{d}$ is the arithmetic mean of pairwise distance between gRNA and target across the entire 20-mer and *n* represents the number of mismatches between the target and the gRNA. This value ranges from 1, in the case of a perfect match, to 0 in the case of a complete mismatch.

This exhaustive search ensures that sequences with small InDels relative the HXB2 reference, outside of the target area, are not penalized. Since the number of overlapping patient HIV-1 sequences in LANL changes by region, the number of sequences evaluated for each gRNA was included in the Supplementary File 1. Due to the nature of the MIT scoring function, it can only process 20 bp protospacers. In the 69 instances where the protospacers were greater than 20 bp they were trimmed to include only the 20 nucleotides proximal to the PAM prior to analysis and for the 11 instances of shorter protospacers they were appended with Ns. Our analysis does not account for non-canonical PAMs as their effects have not been quantitatively examined in the context of the MIT scoring function. In addition, gRNA sequences and/or gRNA target sites that were not clearly identified in previous publications were not included in this analysis.

### Entropy calculation

Previous attempts at identifying conserved regions in HIV-1 have relied on the use of of determinations of Shannon entropy. This quantitation determination represents the average number of items one would expect to see in a sufficiently large sample. The entropy reported here was calculated using the 20-mer protospacers found in the search step described above. Position-specific information content was calculated as the −log_2_(frequency) for each nucleotide in the binding site. In this context, it is ideal to target nucleotides that are conserved and thus have a low information content.

### MIT-off calculation

Since the MIT CRISPR tool is nearly ubiquitous in CRISPR/Cas9 design, an attempt was made to compare our results with the commonly used web-tool. This was performed by partitioning the HXB2 sequence into 250 bp segments, with 50 bp overlaps, and submitting it in batch mode to the webserver (http://crispr.mit.edu). After processing, the raw data was downloaded and compared to the scores calculated above. Due to the nature of this methodology, only 57 of the 229 published gRNAs could be linked in this way to a 20 bp gRNA.

### Released software

Collecting anti-HIV-1 gRNAs from the 26 papers published so far has been complicated due to inconsistencies in reporting gRNAs to the public. To alleviate this confusion, we have annotated them as the target sequence on the forward strand of the reference sequence along with the PAM to indicate the intended orientation. Supplementary Code has been standardized to this format and the provided package recognizes gRNAs in this form. We believe this will ease confusion of what the exact gRNA sequences was, its directionality, and its intended target site thereby allowing fast implementation into the various vector systems currently in use for CRISPR/Cas9.

To facilitate reproducible science, the bioinformatic analyses have been posted to Github under an open-source MIT License [https://github.com/DamLabResources/hiv-crispr-review]. Utilizing the Jupyter Python Notebook format, we have extensively commented and unit-tested the code. The analysis has been broken up into three parts that includes: (1) a sequence processing script that has been developed to calculate the AS for each gRNA across all LANL sequences and can be modified to process user-defined gRNAs; (2) a merging script that links the outputs of our analysis with those from the MIT webserver; and finally (3) a visualization script that aggregates the output and produces publication quality figures. The online analysis also includes heatmaps like Fig. 2B for all gRNAs examined to date. The analysis also produces several summary statistics and figures that may be of interest to researchers in the field.

### Data availability

All data generated or analyzed during this study are included in this published article (and its Supplementary Information files).

## Electronic supplementary material

Dataset 1

Supplemental File Revised
