# Supplementary material for: Designing broad-spectrum anti-HIV-1 gRNAs to target patient-derived variants
Source: Sci Rep. 2017 Oct 31;7:14413. doi: 10.1038/s41598-017-12612-z (PMC5663707; doi:10.1038/s41598-017-12612-z)
Supplement: Supplementary file 2 — Supplemental File Revised [file 41598_2017_12612_MOESM2_ESM.pdf]

Title:

Designing broad-spectrum anti-HIV-1 gRNAs to target patient-derived variants

Will Dampier[1,2,3, #], Neil T. Sullivan[1,2, #], Cheng-Han Chung[1,2], Joshua Chang Mell[4,5], Michael R. Nonnemacher[1,2], Brian Wigdahl[1,2,6,*]

[1]Department of Microbiology and Immunology, Drexel University College of Medicine, Philadelphia, PA, USA, [2]Center for Molecular Virology and Translational Neuroscience, Institute for Molecular Medicine and Infectious Disease, Drexel University College of Medicine, Philadelphia, PA, USA, [3]School of Biomedical Engineering and Health Systems, Drexel University, Philadelphia, PA, USA, [4]Center for Genomic Sciences, Institute for Molecular Medicine and Infectious Disease, Drexel University College of Medicine, Philadelphia, Pennsylvania, USA, [5]Center for Advanced Microbial Processing, Institute for Molecular Medicine and Infectious Disease, Drexel University College of Medicine, Philadelphia, Pennsylvania, USA, [6]Sidney Kimmel Cancer Center, Thomas Jefferson University, Philadelphia, PA, USA

[#]These authors contributed equally to this work

*Corresponding author
Brian Wigdahl, Ph.D., Professor and Chair
Department of Microbiology and Immunology
Director, Institute for Molecular Medicine and Infectious Disease
Drexel University College of Medicine
245 N. 15th St.
Philadelphia PA 19102
Telephone Number: (215) 762-8399
Fax Number: (215) 762-1955
E-mail address: bw45@drexel.edu

Contact Information for other authors:
Will Dampier: wnd22@drexel.edu
Neil T. Sullivan: nts42@drexel.edu
Cheng-Han Chung: cc3423@drexel.edu
Joshua Chang Mell: jcm385@drexel.edu
Michael R. Nonnemacher: mrn25@drexel.edu

Supplementary Information:

Supplementary Code 1: Uploaded package of the on-target anti-HIV-1 gRNA pipeline. In order to facilitate reproducible science, an open source Jupyter notebook has been uploaded to a public github repository [https://github.com/DamLabResources/hiv-crispr-review]. This package contains all data, code, and unit-tests to generate the analyses described in the manuscript.

Supplementary File 1: **Effectiveness of current anti-HIV-1 gRNAs to date across the entire HIV-1 genome for eradication therapies.** All published anti-HIV-1 gRNAs sequences (gRNAs published as of 07/31/2017) were analyzed by the on-target gRNA pipeline. The table includes the reference for that gRNA, the gRNA name, sequence, percentage of sequences cleaved according to the on-target pipeline analysis (percent cleaved, activity score cutoff = 0.75), Shannon entropy of the gRNA target region, and number of sequences evaluated. The MIT-off for gRNAs was determined using http://crispr.mit.edu. See attached excel file for the full analysis.
